# Corpus Callosotomy in 3 Cavalier King Charles Spaniel Dogs with Drug-Resistant Epilepsy

**DOI:** 10.3390/brainsci11111462

**Published:** 2021-11-04

**Authors:** Rikako Asada, Satoshi Mizuno, Yoshihiko Yu, Yuji Hamamoto, Tetsuya Anazawa, Daisuke Ito, Masato Kitagawa, Daisuke Hasegawa

**Affiliations:** 1 Laboratory of Veterinary Radiology, Nippon Veterinary and Life Science University, Musashino 180-8602, Japan; r-asada@shinkei.com (R.A.); edymizuno1020@gmail.com (S.M.); yoshi.yu.vet@gmail.com (Y.Y.); 2 Veterinary Medical Teaching Hospital, Nippon Veterinary and Life Science University, Musashino 180-8602, Japan; y-hamamoto@nvlu.ac.jp; 3 Neurology and Neurosurgery Service, Japan Animal Referral Medical Center Nagoya, Nagoya 468-0003, Japan; tetsuya.anazawa@jarmec.jp; 4 Laboratory of Veterinary Neurology, Department of Veterinary Medicine, College of Bioresource Sciences, Nihon University, Fujisawa 252-0880, Japan; itou.daisuke@nihon-u.ac.jp (D.I.); kitagawa.masato@nihon-u.ac.jp (M.K.); 5 The Research Center for Animal Life Science, Nippon Veterinary and Life Science University, Musashino 180-8602, Japan

**Keywords:** corpus callosotomy, dog, drug-resistant epilepsy, epilepsy surgery, veterinary

## Abstract

Corpus callosotomy (CC) is an established palliative surgery for human patients with drug-resistant epilepsy (DRE), especially those with generalized seizures and multiple or unknown epileptogenic focus. However, there are no reports to describe CC in canine patients with epilepsy. Three client-owned Cavalier King Charles Spaniels with DRE are included in this case series. In presurgical evaluations, an apparent epileptogenic zone was not detected in each dog and CC was conducted. Total CC was performed in one dog, whereas the other two received partial CC. One dog recovered from surgery without any complications, but died suddenly by an unknown cause at 10 h after surgery. For the other two dogs, postoperative evaluations including seizure outcomes, complications, and quality of life of the dogs and owners were assessed for at least 12 months. Both dogs showed a remarkable decrease in seizure frequency (averaged 80.3% reduction) and severity after surgery. The antiseizure medications were maintained, and not only the mentation and activity of the dogs, but also the quality of life of dogs and owners were improved postoperatively. Although technical improvement and more large-scale studies are needed, CC is a treatment option for dogs with DRE in veterinary medicine.

## 1. Introduction

Surgical treatment for drug-resistant epilepsy (DRE) is established in human medicine and has achieved a fair to good prognosis. Corpus callosotomy (CC) is the most common and well-established method of disconnection surgery and is applied mainly as a palliative treatment to reduce the frequency and severity of generalized seizures or focal to bilateral tonic-clonic seizures with unidentifiable or multifocal epileptogenic zone(s) [1,2,3,4].

Epilepsy surgery, including CC, has been poorly reported in veterinary medicine, with only a few experimental and anecdotal cases reported even after 2000 [5,6,7]. Most intracranial surgeries performed in the past as part of the treatment for epileptic seizures lacked evidence from electroencephalography (EEG), magnetic resonance imaging (MRI), or both. In 1995, Bagley et al. reported an experimental study on the surgical procedure and short-term complications of CC in normal beagle dogs [5], but there have been no reports of CC in clinical cases. In the past 20 years, advanced diagnostic and neurosurgical modalities have become widely available in veterinary medicine, and intracranial surgery has become a part of routine clinical practice. Recently, a review of methods for identifying epileptogenic zones in dogs and cats was published [8], and the expectation of epilepsy surgery for dogs and cats with DRE has increased from veterinary surgeons and pet owners [9].

In this case series, we report on CC and its one-year outcome in three Cavalier King Charles Spaniel (CKCS) dog patients affected by DRE.

## 2. Summary of the Clinical Trial of Epilepsy Surgery in Dogs

This case series, as a clinical trial, is a part of the project “Development of epilepsy surgery in small animal veterinary medicine” funded through a Japanese national grant (JSPS KAKENHI grant number 17H01507). The veterinary epilepsy surgery team (VEST) consisted of four board-certificated veterinary neurology specialists and one veterinary neuropathologist from four institutions, in collaboration with human medical doctors specialized in epileptology and epilepsy surgery. In this project, canine patients suspected with DRE and owners seeking epilepsy surgery were presented to VEST and determined to be “true” DRE or not. Inclusion criteria and the definition of DRE for epilepsy surgery are described in Supplementary File S1. Once the case with DRE was approved, VEST members determined additionally required presurgical evaluations and prospective surgical technique for each patient. The three cases included in this paper were approved as DRE, and CC was considered the best option for each dog. To obtain informed consent, each owner of the approved patients was explained the purpose and plan of the study, funding, expected advantages and disadvantages of the CC and other surgical techniques, risk of surgery, follow-up schedules, and necropsy when the patient dies from any causes. This clinical study was approved by the Institutional Review Board of the Veterinary Medical Teaching Hospital of Nippon Veterinary and Life Science University (#H30-1).

In the three cases, each owner was required to record seizures in a diary and to submit a visual-analog scale (VAS) assessment of various items, including the dog’s and owner’s QoL before surgery and at postoperative 3, 6, and 12 months after surgery (Supplementary File S2). The attending doctor also rechecked seizure outcome and follow-up neurological status, scalp encephalogram (EEG), and magnetic resonance imaging (MRI) at postoperative 3, 6, and 12 months. Additionally, antiseizure medications (ASMs) were not allowed to be changed between before and after surgery (until 12 months after surgery), unless the seizures deteriorated, in order to avoid the bias of ASMs.

## 3. Case Presentation

Before participating in the clinical trial, all dogs had undergone the following examinations recommended by the International Veterinary Epilepsy Task Force (IVETF) consensus, which is the standard for the diagnosis of epilepsy in veterinary medicine (Supplementary File S1) [10]: general physical and neurological examination, blood tests, urinalysis, MRI of the brain, cerebrospinal fluid (CSF) analysis, and scalp EEG under sedation. A detailed history of each case is described in Supplementary File S3 and summarized in Table 1.

### 3.1. Case 1

Case 1 was a neutered female CKCS (weight, 6.8 kg) that underwent two surgeries. The patient was 2 years and 9 months old at the first surgery and 3 years and 6 months old at the second. The seizure onset was at 2 months old. Seizure types were facial myoclonic seizures with salivation and right forelimb clonus, which often evolved into generalized tonic-clonic seizures (GTCS). Preoperative seizure frequencies were approximately 15 seizures/day (sz/d), and seizure duration was approximately 10 s (facial myoclonic seizures) to 5 min (when evolved into GTCS). Seizures tended to be severe and she experienced cluster seizures (CS) at 11 months old. At this time, the patient’s activity declined and the dog mainly slept during the day. Neurological examination during the interictal period revealed severe somnolence (sedation) and reduction of postural reactions in all limbs. Left hippocampal atrophy had been noted since the patient was 3 months old and progressed over time, and the ventral hippocampus was hardly visible on MRI before surgery. At the age of 2 years and 7 months, the patient developed super-refractory status epilepticus (SE) (bilateral facial myoclonic seizures). Euthanasia was considered, but treatment was continued at the owner’s request, and, finally, the patient was weaned from a therapeutic coma at 120 h. Since then, the dog had been refractory to multiple ASMs with phenobarbital (PB), zonisamide (ZNS), potassium bromide (KBr), levetiracetam (LEV), and pregabalin (PGB). As soon as the epilepsy surgery trial started, the owners applied for entry.

### 3.2. Case 2

Case 2 was a neutered female CKCS (weight, 7.2 kg) that underwent surgery at 4 years and 10 months old. Seizure onset was at 2 years and 3 months old. The type of seizure was varied, with GTCS, bilateral facial and generalized myoclonic seizures, and circling observed. Seizure frequency was approximately 37 sz/d. Seizure duration was 2 min to <10 min for each seizure type, but once a day the patient presented SE lasting 40 min. Neurological examination in the interictal period revealed severe somnolence and reduction of postural reactions in all limbs. During the daytime, the dog was strongly sedated, slept most of the time, and could only stand and eat or drink for a short time. At the age of 4 years and 1 month, the patient was referred to a secondary care facility and ASM was adjusted by neurologists. However, the dog was refractory to ASMs with PB, ZNS, KBr, LEV, and gabapentin (GBP). The owner entered the clinical trial because seizure control by further ASMs seemed to be unrealistic and the dog’s QoL was clearly decreased.

### 3.3. Case 3

Case 3 was a neutered male CKCS (weight, 10.2 kg) that underwent surgery at 5 years and 5 months old. The dog was found to have mild mitral regurgitation, but was under untreated observation. Seizure onset was at 3 years 4 months old. The type of seizure varied, with GTCS, bilateral facial and generalized myoclonic seizures, and drop attacks (atonic or negative myoclonic seizures) observed. Seizure frequency was approximately 28 sz/d and usually lasted 1 min to <10 min. Neurological examination during the interictal period revealed severe somnolence and reduction of postural reactions in all limbs. The dog had generalized ataxia, decreased consciousness, and could not be active at all during the daytime. He was noticeably polyphagic with impaired cognition, and often tried to eat the owner’s hand when offered to him. Cluster seizures and SE occurred at the age of 5 years and 3 months, and ASM adjustment had been performed. However, the dog was refractory to PB, ZNS, KBr, LEV, and PGB. When the dog was 5 years and 4 months of age, the owner decided to enter the trial.

## 4. Presurgical Evaluations and Strategy for Surgery

Detail explanations of the method for each presurgical evaluation, i.e., scalp EEG, MRI, CSF analysis, and video-intracranial EEG (ViEEG) are described in Supplementary File S4.

### 4.1. Case 1

**Figure 1:**
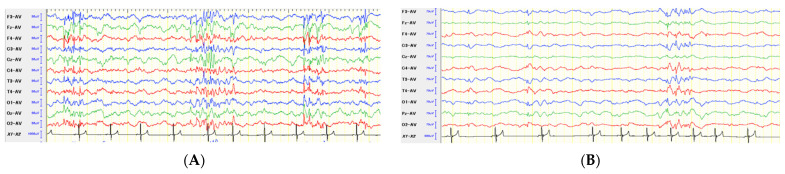
Scalp EEGs before (**A**) and at 12 months after (**B**) the 2nd surgery (anterior 2/3 CC) in Case 1. Generalized and synchronized multiple spike complex with high amplitude was observed frequently preoperatively (**A**). Postoperatively, some spikes were synchronized bilaterally, but spike spread, frequency, and amplitude were improved remarkably (**B**).

**Figure 2:**
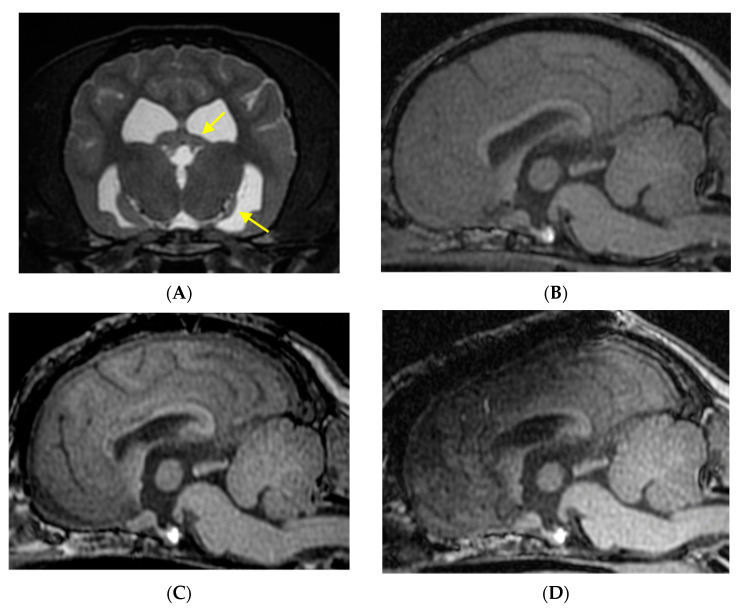
MRIs of preoperative (**A**,**B**), 9 months after the 1st surgery (**C**), and 12 months after the 2nd surgery (**D**) in Case 1. Marked atrophy of the left hippocampus is observed ((**A**) T2-weighted transverse). Predisposed Chiari-like malformation is observed ((**B**–**D**), T1-weighted midline sagittal). Anterior 1/2 CC was performed at the 1st surgery (**C**). An additional caudal extension of the CC (anterior 2/3) and cortical resection of anterior part of the left cingulate gyrus were conducted at the 2nd surgery ((**D**); because of titanium plate on the skull, the signal intensity of the dorsal part of the head is blurred).

Interictal scalp EEG showed bilateral synchronized epileptiform discharges (EDs) (Figure 1A). As mentioned above, this case had progressive left hippocampal atrophy on MRI (Figure 2A) since 3 months. Hippocampal volumetry, performed before surgery at 2 years 4 months old, showed that the left and right hippocampal volumes were 0.10 and 0.28 cm^3^, respectively. Other than left hippocampal atrophy, there were no abnormal or specific findings on each MRI sequence (Figure 2B). Although Chiari-like malformation (CLM) was observed, CLM is a common inherited cranio-cervical abnormality in this breed (CKCS) and is not related to epileptogenicity directly [11]. In this case, although the left hippocampal abnormality was considered an epileptogenic lesion, we were unable to insert depth electrodes into the extremely atrophied hippocampus or perform hippocampectomy. In addition, scalp EEG showed fronto-central dominant spikes, thus we suspected the medial surface of the left frontal lobe or cingulate gyrus as the epileptogenic zone and the hippocampal atrophy as a dual pathology. Therefore, we planned initial intraoperative electrocorticogram (ECoG) of those areas, followed by focal cortical resection or multiple subpial transection (MST) according with ECoG findings. If focal resection or MST failed to suppress EDs intraoperatively, we planned an anterior CC.

### 4.2. Case 2

**Figure 3:**
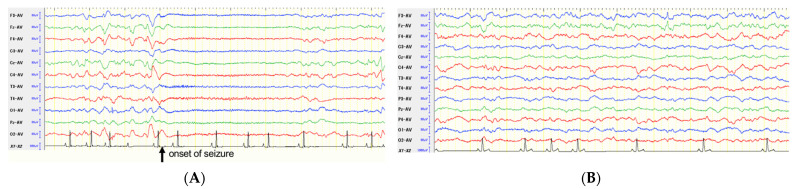
Scalp EEGs before (**A**) and at 12 months after (**B**) total CC in Case 2. Preoperatively, the large sharp wave and following low amplitude and fast rhythmic activities with clinical facial myoclonic seizures (arrow) were observed frequently, other than the common interictal epileptiform discharges during the EEG recording (**A**). On the postoperative EEG, those seizure activities were not observed, and common spikes or sharp waves were sporadically observed (**B**).

**Figure 4:**
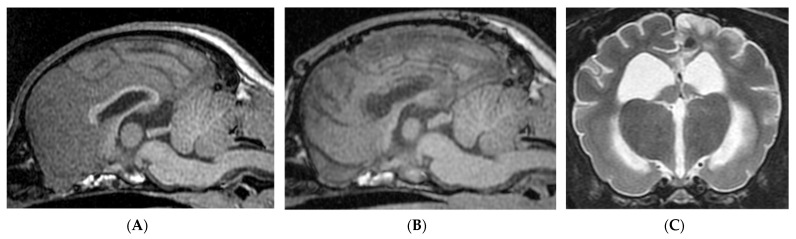
Preoperative (**A**) and postoperative 12 months (**B**,**C**) MRIs in Case 2. Predisposed Chiari-like malformation was observed ((**A**,**B**) midline sagittal T1-weighted). Total CC was performed in this case (**B**), but hemosiderosis was observed around the vein of the corpus callosum. In this dog, however, delayed focal infarction in the cingulate to marginal gyrus of the left parietal lobe was observed at 6 months after surgery ((**C**) transverse T2-weighted).

Scalp EEG showed interictal bilateral synchronized EDs and generalized seizure activities with facial myoclonus (Figure 3A). Other than CLM, there were no abnormal or specific findings on each MRI sequence (Figure 4A). In this case, focal to bilateral tonic-clonic seizures were suspected, and we planned ViEEG monitoring to decide the laterality of the epileptogenicity.

On the ViEEG, however, interictal EDs were bilaterally synchronous, and ictal ECoG with bilateral focal onset evolving GTCS were recorded (Video S1). Therefore, we were unable to determine the laterality of the seizure-onset zone. Thus, we planned to perform total CC.

### 4.3. Case 3

Scalp EEG showed interictal bilateral synchronized EDs and generalized seizure activities with facial myoclonic seizures similar to those in Case 2. Other than CLM, there are no abnormal or specific findings on each MRI sequence. In this case, because the epileptogenic focus was not identified and drop attacks were frequently observed, we planned to perform total CC.

## 5. Surgery

Anesthesia, vital monitoring, fluid therapy, perioperative medications such as analgesics and antibiotics, and a detailed surgical procedure of intraoperative ECoG and CC are described in Supplementary File S5. Additionally, an operatory movie of CC (from Case 2) is presented as Video S2.

### 5.1. Case 1

At first, intraoperative ECoG was measured on the area of the cruciate sulci (counterpart of the central sulcus in humans). Epileptiform discharges were synchronized bilaterally, but the amplitude of spikes in the left hemisphere was higher than that in the right. After that, ECoG was also measured from the medial surface of interhemispheric fissure of each side (i.e., bilateral supplementary motor cortexes and cingulate gyri), and recorded spikes were clearly higher than those from the cruciate sulci and those in the left were more remarkable (Figure S2A in Supplementary File S4). Therefore, we performed MST in those cortexes of the left hemisphere. After MST, however, EDs persisted and remained synchronized bilaterally. Thus, we performed an anterior 1/2 (the genu and a rostral part of the body) CC (Figure 2C). Post-CC EcoG revealed a partial desynchronization of spikes (Figure S2B in Supplementary File S4).

After surgery, all seizures disappeared for 3 months. However, seizures recurred with increased seizure frequency at the next 6 months. Seizure frequency at the postoperative 9 months returned equally to that before surgery. We performed ViEEG monitoring around the left cingulate gyrus and investigated the extent of resection based on the distribution of EDs during facial myoclonic seizures. Therefore, as a second surgery, we performed additional cortical resection in the left cingulate gyrus and caudally extended the CC, resulting in an anterior 2/3 CC (Figure 2D). Seizure outcome of the second surgery is described in the next Section 6.1.

### 5.2. Case 2

At first, intraoperative pre-CC ECoG was measured on the area overlying the parietal lobe for each hemisphere. Epileptiform discharges were synchronized bilaterally (Figure S2C in Supplementary File S4). Based on the results of ViEEG and intra-operative ECoG, we performed total CC (Figure 4B). Post-CC ECoG revealed partial desynchronization of spikes (Figure S2D in Supplementary File S4). In Case 2, transient cognitive dysfunction was observed postoperatively; the patient did not respond to calls and exhibited wandering behavior for one week after surgery.

### 5.3. Case 3

As in Case 2, pre-CC ECoG was measured on the area overlying the parietal lobe for each hemisphere. Epileptiform discharges were synchronized bilaterally (Figure S2E in Supplementary File S4). Although we attempted a total CC, we could not bisect the genu due to intraoperative brain swelling. However, intraoperative post-CC ECoG showed partial desynchronization of EDs (Figure S2F in Supplementary File S4), so the surgery was ended.

The dog recovered from surgery uneventfully. However, a sudden decrease in consciousness and cardiopulmonary arrest occurred at 10 h after awaking from anesthesia, and the dog died despite resuscitation. With the owner’s consent, systemic autopsy was conducted to determine the cause of death. Pathologically, although hemorrhage and neutrophilic infiltration was observed in the corpus callosum and cingulate gyrus at the surgical site, mild mitral valve degeneration of the heart, congestion of the lungs and liver, and pulmonary edema were observed. There were no specific abnormalities in the brain other than the surgical lesions and no direct cause of the circulatory and respiratory disturbances was identified.

## 6. Results and Follow-Up Evaluations

Because Case 3 was lost as mentioned above, follow-up evaluations were obtained from the two other cases. Case 1 underwent surgery twice, and the follow-up data described below are those after the second surgery. The averaged total (including all seizure types) seizure reduction rates of the two surviving dogs at 3, 6, and 12 months after surgery were 76.6, 75.9, and 88.4%, respectively. For GTCS, seizure reduction rates for each time point were 76.6, 53.0, and 43%, respectively, while those of facial myoclonic seizures were 78.7, 74.8, and 88.6%, respectively. Reduction rates of each case (Table 1) and other postoperative information are described below.

### 6.1. Case 1

#### 6.1.1. Seizure Outcome

Postoperative seizure frequencies of focal and generalized seizures were reduced in both the first and second surgeries. The dog was seizure-free for 3 months after the first surgery. Thereafter, the seizure frequency increased and reached the same level as before surgery (15 sz/d) at 9 months after the first surgery. However, most of the postoperative seizures were mild compared to preoperative ones, with small bilateral facial twitching lasting less than 10 s.

After the second surgery, the seizure frequency continued to decrease slowly. At 6 months after the second surgery, the seizure frequency increased slightly once, but continued to decrease to 1.23 seizures/day at 1 year after surgery. Seizure reduction rates at 3, 6 and 12 months after second surgery (including all seizure types) were 61.8, 64.2 and 76.7%, respectively. Postoperative seizures were mostly limited to the right side of the body (left hemisphere onset) and the duration of seizures was usually about 10 s. At 2 years and 2 months after the second surgery (at the time of writing this report), seizure frequency was 1.0 sz/d (93.4% reduction).

#### 6.1.2. Postoperative Neurological Status and Complications

The dog’s consciousness and activity were clearly improved after both surgeries. During the seizure reduction period after the first and second surgery, the patient showed positive behavior, running around and playing with toys with her owners, while she was deeply somnolent and could not run and play before surgery. The owners reported that the dog’s activity had recovered to that before the epilepsy onset.

No serious postoperative complications were observed. Only mild ataxia with some slipping at the start of running was observed. During the entire observation period from the first surgery, neurological examination revealed a mild decrease of conscious proprioception in the right hind limb.

#### 6.1.3. Follow-Up Scalp EEG and MRI

At 3 months after the second surgery, the EEG findings improved. Most EDs were asynchronous and localized to the left frontal region, and the frequency and amplitude of the spikes decreased compared with the preoperative findings. Still some multiple spikes with high amplitude were synchronized bilaterally, isolated, and small asynchronous spikes were also observed at 6 and 12 months after the second surgery (Figure 1B).

There was no evidence of hemorrhage or infarction around the CC and cortical resection areas (Figure 2D). Hippocampal atrophy progressed further, and slight atrophy of the right hippocampus was also observed. Hippocampal volumetry was performed at 1 year after the second surgery (4 years 6 months old), the left and right hippocampal volume was 0.08 and 0.25 cm^3^, respectively.

#### 6.1.4. Visual-Analog Scale by the Owners

After the second surgery, the VAS gradually improved (Supplementary File S2). Both the frequency of seizures and daily activeness were improved compared with the preoperative values. The QoL of the dog improved by 62 points and that of the owner improved by 45 points. At 1 year after surgery, owners reported a high level of satisfaction with the epilepsy surgery.

### 6.2. Case 2

#### 6.2.1. Seizure Outcome

Postoperative seizure frequency was greatly reduced. The seizure reduction rates at 3, 6 and 12 months after surgery (including all seizure types) were 91.3, 87.6 and 100%, respectively. In addition, most of the postoperative seizures became mild, with small bilateral facial twitching observed for less than 10 s to 1 min. Status epilepticus that was seen every day before surgery had disappeared, and facial myoclonic SE was seen only once at 6 months after surgery. The patient achieved seizure-free status for 3 months from 9 months to 1 year postoperatively.

#### 6.2.2. Postoperative Neurological Status and Complications

The consciousness and activity of the patient was clearly improved after the surgery. Signs of cognitive dysfunction observed after surgery had resolved within 1 week. From the first month after surgery, the owner reported improvement in the dog’s activity and cognitive function; she remained awake in daytime, took walks, and played with the owners.

No serious postoperative complications were observed. In the neurological examination during the postoperative hospitalization, a mild decrease in postural reactions of the right limbs (hemiparesis) was observed, but this had improved by 3 months after surgery.

#### 6.2.3. Follow-Up Scalp EEG and MRI

Postoperatively, the EEG findings gradually improved. At 3 months postoperative follow-up, although bilateral synchronized EDs with facial myoclonus were observed similar to preoperative EEG, small spikes localized to the left frontal region, were observed. At 6 months after surgery, the facial myoclonus and EDs were localized to the left hemisphere. At 12 months after surgery, the EEG findings were clearly improved, no seizures were observed during EEG recording, and the frequency of spikes was minimal and localized to the left frontal region (Figure 3B).

Follow-up MRI at 3 months after surgery revealed total CC had been completed, but also showed a small necrotic lesion in the subcortical white matter of the left marginal gyrus and a hemosiderin deposit along the vein of the corpus callosum. On the follow-up MRI at postoperative 6 months, an extended lesion of the suspected delay-onset infarction in the left parietal lobe was observed and was considered to be the responsible lesion of facial myoclonic SE, which was observed only once at that time. At 1 year after surgery, there was no improvement in these MRI findings (Figure 4B,C).

#### 6.2.4. Visual-Analog Scale by the Owners

After surgery, the VAS gradually improved (Supplementary File S2). Both the frequency and activity of each seizure were improved compared with the preoperative values. The QoL of the patient dog increased by 78 points and the owner’s increased 74 points. At 1 year after surgery, owners reported a high level of satisfaction with the epilepsy surgery.

## 7. Discussion

This is the first report describing CC and its 1-year outcome in canine patients with DRE. In this report, we investigated various presurgical evaluations to detect the epileptogenic zone using currently available veterinary diagnostic modalities [8,10], and applied CC to patients with primary and secondarily generalized seizures and in whom the epileptogenic zone could not be identified. Although one dog died immediately after surgery, CC in the two surviving dogs showed not only remarkable reductions in seizure frequency and severity, but also improvement of mentation, activity, and QoL. Additionally, the owners of those dogs reported that their own QoL had been improved and their satisfaction with CC for their dogs was high.

In human medicine, the seizure reduction rate of CC varies depending on the separation amount of the corpus callosum and the seizure type of the patients, and few patients (0–10%) achieve complete seizure freedom [1,2,3,4,12]. However, an improvement of patients’ daily functions has been observed in many patients, especially in children, and improvement of QoL of patients and their families have also been reported [1,3,12]. This is thought to result from a decrease in seizure frequency and a reduction in the effects of ASM, as well as (re-)development/organization in brain function or network after the surgery. In this canine case series, an improvement of activity and cognitive function (i.e., being able to enjoy a dog’s s life by taking walks and playing) was observed without the reduction of ASMs. Thus, the improvement of QoL may be related to the relief of seizures by CC. The improvement in QoL of the patient dogs had also led to the improvement in QoL of their owners; this is an important point for considering human and animal welfare. Thus, the success of CC has a positive impact on owners who suffer from the frequent seizures and related QoL decline of their dogs’.

In both survived cases of this study, CC could reduce seizure frequency markedly (80.3% reduction in total) regardless of seizure type. For dog’s owners, decreasing GTCS was most impressive and favorable; however, on the objective seizure counts, reduction rate of GTCS was 57.5% while that of myoclonic seizures was 80.7%. In human medicine, CC is most effective against “drop attacks”, which approximate ≥80% pediatric patients undergoing CC show Engel class I–II seizure outcome [1,2]; but it lessens in adult cases [3] and/or other seizure types [1,2,3,4]. In this canine case series, drop attack was only observed in Case 3 who died immediately after surgery, so that we could not state the effect of CC on drop attacks in dogs. The reduction rates of GTCS and facial myoclonic seizures in two survived dogs were similar or seemed somewhat superior to those in human adults [1,4]. However, it is too premature to compare with humans’ results, and future case accumulation and large-scale investigation are needed to describe the efficacy of CC in canine epilepsy.

Lateralization of the EDs was observed in the postoperative EEG in the two surviving dogs, which suggests that a rapid generalization from focal onset seizures or apparent (ostensible, pseudo) generalized seizures in dogs would be clarified as focal seizures by performing CC. Postoperative lateralization of EDs and/or unilateralization of seizure symptoms after CC has been reported as an advantage of CC in human medicine and suggests the possibility of detecting epileptogenic zones and the opportunity for further resection surgery [13,14]. We do not anticipate a second (third for Case 1) resection surgery for the dogs in this report at present, but the possibility of further resection would be considered if the owner requested such in the future.

Because the neuroanatomical or functional connectivity of the epileptic brain in dogs has not been studied sufficiently, and total bisection of the corpus callosum has been reported not to effect canine behavioral or neurological status such as disconnection (or split-brain) syndrome in humans during previous experimental study of CC [5], we attempted total CC for all three canine patients in this report. As a result, however, total CC was achieved in only one (Case 2) of the three patients. In two dogs, the genu (Case 3) or splenium (Case 1) of the corpus callosum had remained, which may be caused by the small size of the canine head, as well as a narrow surgical field that prevents visualization of the end portion of the corpus callosum. In dogs, as well as human beings, there are branches of the anterior cerebral artery on the rostral side of the corpus callosum, while on the caudal side there are the veins of the corpus callosum and the internal cerebral vein, both of which are confluent to the great cerebral vein (vein of Galen). In this report, we attempted visually (under the surgical microscope) to dissect the corpus callosum from the interhemispheric fissure, because damage to these vessels may cause massive hemorrhage and surgical operation with inadequate vision may be dangerous. However, transient and slight hemiparesis of the contralateral side to the retracted hemisphere and focal hemorrhage and infarction (to necrosis) around the CC site were observed in the two surviving dogs. This was thought to be due to invasion by retracting the hemisphere during approach to the corpus callosum, as well as by the dissection of the adhesions between bilateral cingulate gyri. It has been reported that it was necessary to detach the adhesions around the cingulate gyrus in a previous experimental CC in dogs [5] and humans [2,15]. In the present cases, it was also thought that these manipulations damaged the surrounding vessels and caused the hemorrhages and infarction. Therefore, to reduce the surgical complications and to improve the dissection area, more careful surgical procedures, such as gentle retraction of the hemisphere and/or cautious detaching of the cingulate gyri, and a more extended surgical field (in cases of one-stage total CC) would be required. On the other hand, a transient (one-week) impairment of cognitive function was observed after surgery in Case 2, which achieved total CC. This has not been reported in dogs that underwent anterior CC in this study or in normal dogs after experimental CC [5]. At this time, we are unable to determine whether this transient cognitive dysfunction corresponds to the postoperative disconnection syndrome observed in humans after CC. Therefore, the surgical complications of canine CC should be investigated in the future with a larger number of cases. However, there is another question raised as to whether total CC is necessary or whether partial CC, similar to human anterior CC, is sufficient for canine patients with DRE. This will also be resolved by accumulating additional cases.

No apparent cause of death was identified for Case 3 that died postoperatively. The patient died suddenly without any apparent epileptic seizure. Although the death occurred soon after the surgery, the mortality rate of CC in dogs had been reported as low [5]. Since total necropsy and pathological findings could not reveal an apparent causal relationship between the surgery and the death in this dog, the possibility of unexpected sudden death in epilepsy patients (SUDEP) cannot be ruled out. Currently, in veterinary medicine, it has been recognized that SUDEP can occur in dogs, as well as humans [16]. This dog was suffering from GTCS several times daily before the surgery, and seizure control was not achieved despite using multiple and high-dose ASMs. Although the risk factors for SUDEP in dogs is still under debate, the characteristics of this case are similar to those of SUDEP in humans. In recent years, cardiac dysfunction in epileptic patients has been studied in humans, and the concept of the “epileptic heart” has been proposed [17,18]. This indicates that surges in catecholamines and hypoxemia leading by recurrent epileptic seizures can cause myocardial damage, leading to electrical and mechanical dysfunction. Although the pathological findings showed no evidence of myocardial dysfunction, the patient had a mild mitral regurgitation (which is very common in adult CKCS) as an underlying disease. Therefore, the repeated severe preoperative seizures, a long anesthesia, and surgery may have stressed the ill heart and caused cardiac dysfunction. Further studies on SUDEP and cardiac function assessment need to be conducted in canine epilepsy patients.

## 8. Conclusions

While there are many limitations to describing the utility and efficacy of CC in dogs with DRE from our small case series, and improvement of surgical techniques are needed, our results suggest that CC can reduce intractable seizures markedly and improve the QoL of both dogs and owners. Therefore, CC is a treatment option for canine patients with DRE in veterinary medicine. The authors hope that this report might provide new hope for many dogs suffering from DRE and for their owners.

## Figures and Tables

**Table 1:** Summary of pre- and post-operative status in each patient.

	Case 1		Case 2	Case 3
Dog breed (color)	CKCS (tricolor)		CKCS (Blenheim)	CKCS (Blenheim)
Sex	Neutered female		Neutered female	Neutered male
Age at initial Sz	2 m		2 y 3 m	3 y 4 m
Age at op	2 y 9 m	3 y 6 m	4 y 10 m	5 y 5 m
Preop Sz types	- Bi facial Myocl sz with R forelimb clonus - GTCS	- Bi or R facial Myocl sz - GTCS	- Bi facial or G Myocl sz - GTCS - circling (running fit)	- Bi facial or G Myocl sz - GTCS - drop attack (atonic sz)
Preop Sz frequency (sz/d)	15	15.1	37.2	28.3
Preop Sz duration	10 s–5 min	10 s–10 min	2–10 min	1–10 min
Preop CS/SE	CS+/SE+	CS+/SE+	CS+/SE+	CS+/SE+
Preop Neurol findings	- decreased postural reactions in all 4 limbs - G ataxic gait	- decreased postural reactions in R limbs (mild)	- decreased postural reactions in all 4 limbs - G ataxic gait	- decreased postural reactions in all 4 limbs - G ataxic gait
Preop conscious/activity	- somnolence - decreased activity	- somnolence - decreased activity (mild)	- somnolence - decreased activity	- somnolence - polyphagia - impaired cognition - decreased activity
ASM history	ZNS, PB, KBr, LEV, GBP		ZNS, PB, LEV, GBP	ZNS, PB, KBr, LEV, PGB
Preop ASMs	ZNS, PB, KBr, LEV		ZNS, PB, LEV, GBP	ZNS, KBr
EEG findings	Bi synchronized EDs or G sz activities with facial Myocl sz			
MRI findings	L hippocampus atrophy (with CLM)		Non lesion (with CLM)	Non lesion (with CLM)
CSF findings	Normal	Normal	Normal	Normal
ViEEG monitoring	NE	1 week	1 week	NE
Amount of CC	54.3% (genu + body1/2)	70% (+body1/3)	100%	77% (body + splenium)
Op time *	430 min	643 min	710 min	600 min
Postop Sz type	- Bi or R facial Myocl sz - GTCS	- R facial Myocl sz - GTCS (R side main)	- Bi facial Myocl sz - circling	NA
Postop 3 m Sz frequency (sz/d) (Reduction rate)	0.38 (97.5%)	5.77 (61.8%)	3.24 (91.3%)	NA
Postop 3–6 m Sz frequency (sz/d) (Reduction rate)	3.10 (79.3%)	5.4 (64.2%)	4.63 (87.6%)	NA
Postop 6–12 m Sz frequency (sz/d) (Reduction rate)	15.1 (~9 m) (−0.7%)	3.52 (76.7%)	0 (100%)	NA
Postop Neurol findings	decreased postural reactions in R limbs (within 2 weeks)			NA
Postop conscious/activity	improved	improved	improved (transient cognitive dysfunction for 1 week)	NA

CKCS, Cavalier King Charles Spaniel; Sz, seizure; op, operation; preop, preoperative; postop, postoperative; y, years; m, months; sz/d, seizures/day; Bi, bilateral; R, right; G, generalized; Myocl, myoclonic; GTCS, generalized tonic-clonic seizures; CS, cluster seizure; SE, status epilepticus; ASM, antiseizure medication; ZNS, zonisamide; KBr, potassium bromide; PB, phenobarbital; LEV, levetiracetam; GBP, gabapentin; PGB, pregabalin; EEG, electroencephalogram; ED, epileptiform discharge; MRI, magnetic resonance imaging; CLM, Chiari-like malformation; CSF, cerebrospinal fluid; ViEEG, Video-intracranial EEG; NE, not exam; CC, corpus callosotomy; NA, not available. * Op time including anesthesia and postoperative MRI.

## Data Availability

The data presented in this study are available in the article and Supplementary Materials. Further data are available from the corresponding author on reasonable request.

## Supplementary Materials

The following are available online at https://www.mdpi.com/article/10.3390/brainsci11111462/s1. Supplementary File S1, The definition and diagnosis of epilepsy in dogs and inclusion criteria for the clinical trial of epilepsy surgery; Supplementary File S2, Visual-analog scale (including Tables S1–S3); Supplementary File S3, Case histories; Supplementary File S4, Methods of each presurgical evaluation (including Figures S1 and S2) [19]; Supplementary File S5, Surgical procedures; Video S1, ictal video-intracranial EEG in Case 2; and Video S2, Operatory video of corpus callosotomy (Case 2).

## References

[1] Asadi-Pooya A.A., Sharan A., Nei M., Sperling M.R. (2008). Corpus callosotomy. Epilepsy Behav..

[2] Graham D., Tisdall M.M., Gill D. (2016). Corpus callosotomy outcomes in pediatric patients: A systematic review. Epilepsia.

[3] Unterberger I., Bauer R., Walser G., Bauer G. (2016). Corpus callosum and epilepsies. Seizure.

[4] Park M.S., Nakagawa E., Schoenberg M.R., Benbadis S.R., Vale F.L. (2013). Outcome of corpus callosotomy in adults. Epilepsy Behav..

[5] Baglay R.S., Baszler T.V., Harrington M.L., Pluhar G.E., Moore M.P., Keegan R.D., Greene S.A. (1995). Clinical effects of Longitudinal Division of the Corpus Callosum in Normal Dogs. Vet. Surg..

[6] Martlé V., Van Ham L.M.L., Boon P., Caemaert J., Tshamala M., Vonck K., Raedt R., Polis I., Bhatti S. (2016). Vagus Nerve Stimulator Placement in Dogs: Surgical Implantation Technique, Complications, Long-Term Follow-Up, and Practical Considerations. Vet. Surg..

[7] Zilli J., Kressin M., Schänzer A., Kampschulte M., Schmidt M.J. (2021). Partial cortico-hippocampectomy in cats, as therapy for refractory temporal epilepsy: A descriptive cadaveric study. PLoS ONE.

[8] Hasegawa D. (2016). Diagnostic techniques to detect the epileptogenic zone: Pathophysiological and presurgical analysis of epilepsy in dogs and cats. Vet. J..

[9] Jones G.M.C., Volk H.A., Packer R.M.A. (2021). Research priorities for idiopathic epilepsy in dogs: Viewpoints of owners, general practice veterinarians, and neurology specialists. J. Vet. Intern. Med..

[10] De Risio L., Bhatti S., Muñana K., Penderis J., Stein V., Tipold A., Berendt M., Farqhuar R., Fischer A., Long S. (2015). International veterinary epilepsy task force consensus proposal: Diagnostic approach to epilepsy in dogs. BMC Vet. Res..

[11] Driver C.J., Volk H.A., Rusbridge C., Van Ham L.M. (2013). An update on the pathogenesis of syringomyelia secondary to Chiari-like malformations in dogs. Vet. J..

[12] Maehara T., Shimizu H. (2001). Surgical outcome of corpus callosotomy in patients with drop attacks. Epilepsia.

[13] Chen P.C., Messina S.A., Castillo E., Baumgartner J., Seo J.H., Skinner H., Gireesh E.D., Lee K.H. (2020). Altered integrity of corpus callosum in generalized epilepsy in relation to seizure lateralization after corpus callosotomy. Neurosurg. Focus.

[14] Clarke D.F., Wheless J.W., Chacon M.M., Breier J., Koenig M.K., McManis M., Castillo E., Baumgartner J.E. (2007). Corpus callosotomy: A palliative therapeutic technique may help identify resectable epileptogenic foci. Seizure.

[15] Schaller K., Cabrilo I. (2016). Corpus callosotomy. Acta Neurochir..

[16] Huenerfauth E., Nessler J., Erath J., Tipold A. (2021). Probable sudden unexpected death in dogs with epilepsy (pSUDED). Front. Vet. Sci..

[17] Verrier R.L., Pang T.D., Nearing B.D., Schachter S.C. (2020). The Epileptic Heart: Concept and clinical evidence. Epilepsy Behav..

[18] Verrier R.L., Pang T.D., Nearing B.D., Schachter S.C. (2021). Epileptic heart: A clinical syndromic approach. Epilepsia.

[19] Mizoguchi S., Hasegawa D., Kuwabara T., Hamamoto Y., Ogawa F., Fujiwara A., Matsuki N., Fujita M. (2014). Magnetic resonance volumetry of the hippocampus in familial spontaneous epileptic cats. Epilepsy Res..

