# Supplementary material for: Corpus Callosotomy in 3 Cavalier King Charles Spaniel Dogs with Drug-Resistant Epilepsy
Source: Brain Sci. 2021 Nov 4;11(11):1462. doi: 10.3390/brainsci11111462 (PMC8615928; doi:10.3390/brainsci11111462)
Supplement: Supplementary file 1 [file brainsci-11-01462-s001.zip › brainsci-1446869-supplementary/Suppl File S1_1018.pdf]

brain sciences

MDPI

Supplementary File S1.

The definition and diagnosis of epilepsy in dogs and inclusion criteria for the epilepsy surgery clinical trial

1. Diagnostic criteria of epilepsy in dogs

Diagnosis of canine epilepsy in this study was performed according to diagnostic criteria established by the International Veterinary Epilepsy Task Force (IVETF) [1]. Details of these criteria are described in the original consensus report [1]. Briefly, clinical "epilepsy" is defined as having at least 2 unprovoked epileptic seizures >24 h apart. All animals matching this definition are diagnosed with epilepsy. Those patients are then classified into idiopathic or structural epilepsy (excluding reactive seizures) by the diagnostic criteria of canine idiopathic epilepsy (IE), which consists of a 3 tier system for confidence level. For the tier I confidence level for diagnosis, the patient must fulfill the definition of epilepsy, as well as a seizure onset at an age between 6 months and 6 years, unremarkable physical and neurological examinations, and no clinically significant abnormalities on minimum data base of blood tests and urinalysis. In this level, patients with reactive seizures are excluded from epilepsy, and patients with structural epilepsy have not been diagnosed with IE. IE patients diagnosed with tier I level and patients suspected structural epilepsy go to tier II level examinations including fasting and postprandial bile acids, brain MRI, and CSF analysis. Most patients with structural epilepsy would be diagnosed at this level. Patients with drug-resistant epilepsy who want to enter the epilepsy surgery clinical trial are required to be tier II level. Tier III level of confidence level includes scalp EEG (because EEG is uncommon and very specific test in the veterinary field). Therefore, EEG is usually performed by veterinary epileptologists, such as the member of the project team of this study, after entry to the trial.

2. Inclusion criteria for the epilepsy surgery clinical trial

If the owner understands the significance of the clinical trial, is supportive of the trial, and is able to comply with medications and clinic visits, dogs with drug-resistant epilepsy that have one of the following three conditions are eligible. Therefore, all canine patients who underwent epilepsy surgery in this study had been diagnosed with idiopathic or structural epilepsy by the abovementioned criteria and are consistent with any of the following conditions.

2.1 Condition 1: Canine patients with epilepsy who do not respond (> 2 seizures per 3 months) to 3 of the 4 recommended and therapeutic doses of antiseizure medications (ASM) in veterinary medicine listed below: Phenobarbital (PB), potassium bromide (KBr), zonisamide (ZNS), and levetiracetam (LEV).

Maximum permissible dosage or serum concentration (trough) for each ASM;

- PB (trough serum concentration): 30-35 μg/mL
- KBr (trough serum concentration): 2.5-3.0 mg/mL
- ZNS (trough serum concentration): 40-50 μg/mL
- LEV (dosage): 40–60 mg/kg thrice daily

2.2 Condition 2: Epileptic canine patients with severe adverse effects of ASMs and difficulty in maintaining QoL. Patients with epilepsy who are taking multiple ASMs such as PB, KBr, or ZNS, LEV to control seizures, but have difficulty in maintaining QoL due to severe or multiple adverse effects or the underlying disease(s), which is affected by ASMs; and patients in which seizure frequency increases when ASMs are reduced.

Examples;

- Patients with difficulty in daily life due to constant sedation and severe ataxia.
- Patients with an increase in seizures when ASMs are reduced.
- Patients who want to reduce the dosage of ASMs because they have or are developing hepatic or renal damage caused by ASMs, but the reduction of dosage makes seizure control difficult.

2.3 Condition 3: Drug-resistant cases from structural epilepsy without progressive disease; Patients with structural epilepsy diagnosed by MRI and CSF examination who do not have any progressive disease such as brain tumor or

encephalitis; and patients whose only treatment target is seizure control and who match the first two conditions (refractory to 3 out of 4 ASMs or reduced QoL due to ASMs).

The following are examples of conditions that may be targeted;

- Cortical dysplasia (polymicogyria, lissencephaly, schizencephaly, etc.)
- Post-traumatic epilepsy due to ulegyria caused by previous head injury
- Patients who have undergone brain tumor resection surgery in the past and have no recurrence of the tumor, but are left with intractable epilepsy
- Patients diagnosed with idiopathic or infectious encephalitis and had been treated with suitable therapy and are in remission, with no progression of encephalitis for more than one year without any drugs for encephalitis other than ASMs, but with drug-resistant post-encephalitis epilepsy only.

Reference

1. De Risio, L.; Bhatti, S.; Muñana, K.; Penderis, J.; Stein, V.; Tipold, A.; Berendt, M.; Farqhuar, R.; Fischer, A.; Long, S.; et al. International veterinary epilepsy task force consensus proposal: diagnostic approach to epilepsy in dogs. *BMC Vet. Res.* **2015**, *11*, 148, doi:10.1186/s12917-015-0462-1.
