# Supplementary material for: Corpus Callosotomy in 3 Cavalier King Charles Spaniel Dogs with Drug-Resistant Epilepsy
Source: Brain Sci. 2021 Nov 4;11(11):1462. doi: 10.3390/brainsci11111462 (PMC8615928; doi:10.3390/brainsci11111462)
Supplement: Supplementary file 1 [file brainsci-11-01462-s001.zip › brainsci-1446869-supplementary/Suppl File S2_1103.pdf]

brain sciences

MDPI

Supplementary File S2.

Visual-analog scale (VAS)

To evaluate owners' subjective assessment, owners were instructed to record using a visual analog scale (VAS) the "frequency of focal seizures", "frequency of generalized seizures", "frequency of cluster seizures", "frequency of status epilepticus", "daily activeness", "burden of medications on the dog", "appetite", "ataxia", "sedation (level of arousal)", "QOL of the patient", "QOL of the owner/family" and "total satisfaction with the corpus callosotomy". The owner recorded their feeling on a 100 mm line (0= favorable to 100= worst) in each question at before surgery (with informed consent) and at 3, 6, 9, and 12 months. The researcher collected the VAS sheet at each follow-up consultation and measured the distance of mark of each evaluation item.

In Case 1 and Case 2, decreased seizure frequency and an improvement in ataxia and sedation were observed in VAS at 12 months after surgery. In addition, appetite and activity were improved, the QoL of the owner and the patient dog were improved, and the owner reported a high level of satisfaction with the CC at 12 months after surgery. Actual VAS scores and improved percentages from the preoperative to the 1-year follow-up of each case are shown in the Tables S1–S3, respectively.

Visual Analog Scale (ver. 3)

Date：mm/dd/yy ______ （preoperative・postoperative ____ months）

Owner's name：________ Dog's name：________

Please mark a vertical line on the horizontal line at the point which most accurately describes your feelings or impression to each question.

1. Frequency of seizure day

None ←————————————→ High

______ mm

2. Frequency of focal seizures

None ←————————————→ High

______ mm

3. Duration of focal seizures

None ←————————————→ High

______ mm

4. Frequency of Generalized Tonic-Clonic Seizures

None ←————————————→ High

______ mm

5. Frequency of Cluster Seizures

None High

←→ ____mm

6. Frequency of Status Epilepticus (≥ 5 minutes)

None High

←→ ____mm

7. Severity of Sedation (consciousness)

Normal Always Sleeping

←→ ____mm

8. Severity of Ataxia

None Hard to walk and stand

←→ ____mm

9. Degree of Activity/Daily life

Active (same as before the onset of epilepsy) Worst

←→ ____mm

10. Appetite

None Too much

←→ ____mm

11. Burden of medication on your dog

No drugs Burdensome

←→ ____mm

12. The quality of life in your dog

Excellent Worst (considering euthanasia)

←→ ____mm

13. The quality of life in the owner (yourself)

Excellent Worst

←→ ____mm

14. Satisfaction with the surgery

Satisfied ⟷ Regret ________ mm

Table S1. Changes of VAS score in Case 1

	1st surgery				2nd surgery			
Questions (mm)	Pre	Post 3m	Post 6m	Post 9m† (Preop 2nd)	Post 3m	Post 6m	Post 12m	Improved %*
Frequency of focal seizures	95	0	90	100	97	90	54	24.4
Frequency of generalized seizures	65	0	50	75	76	50	28	28.5
Frequency of cluster seizures	96	0	72	100	90	72	35	35.9
Frequency of status epilepticus	96	0	0	10	90	0	6	81.6
Activity level of the dog	84	0	55	50	45	55	20	55.4
Burden of medication on the dog	95	51	90	80	80	90	87	16.1
Appetite of the dog	96	100	62	80	80	62	56	23.6
Ataxia in the dog	69	0	34	47	67	34	6	54.6
Conscious level in the dog	77	13	52	79	68	52	70	27.7
QOL of the dog	74	13	44	60	58	44	12	48
QOL of the owner	57	15	53	30	50	53	12	37.7
Satisfaction with surgery	NA	0	6	29	50	6	5	84

Values are actual measurements (mm) except the improved percentage. Pre, preoperative; Post 3, 6, (, 9) and 12 m; postoperative months; QOL, quality of life; NA, not applicable. * Improved percentage (%) = 100- {(average of 6 postoperative values) × 100 / preoperative (1st surgery) value}. † Postoperative 9 months of the 1st surgery was considered to the preoperative values of 2nd surgery.

Table S2. Changes of VAS score in Case 2

Questions (mm)	Pre	Post 3m	Post 6m	Post 12m	Improved %*
Frequency of focal seizures	85	17	10	4	87
Frequency of generalized seizures	73	18	5	17	60.7
Frequency of cluster seizures	95	3.2	5	15	99.9
Frequency of status epilepticus	46	7.5	0	3	99.8
Activity level of the dog	67	17	7	5	61.7
Burden of medication on the dog	96	96	95	95	0.69
Appetite of the dog	86	56	49	50	39.9
Ataxia in the dog	29	35	34	20	-2.3
Conscious level in the dog	70	25	20	5	76.2
QOL of the dog	83	5.4	15	5	89.8
QOL of the owner	81	4.3	13	7	73.5
Satisfaction with surgery	NA	2.2	0	1	96.8

Values are actual measurements (mm) except the improved percentage. Pre, preoperative; Post 3, 6, and 12 months; postoperative months; QOL, quality of life; NA, not applicable. * Improved percentage (%) = 100- {(average of 3 postoperative values) × 100 / pre-operative value}.

Table S3. Changes of VAS score in Case 3

Questions (mm)	Pre
Frequency of focal seizures	80
Frequency of generalized seizures	45
Frequency of cluster seizures	98
Frequency of status epilepticus	48
Activity level of the dog	95
Burden of medication on the dog	75
Appetite of the dog	67
Ataxia in the dog	80
Consciousness level in the dog	90
QOL of the dog	71
QOL of the owner	95
Satisfaction with surgery	NA

This case died immediately after the surgery, therefore, only preoperative VAS is available.
