# Supplementary material for: Corpus Callosotomy in 3 Cavalier King Charles Spaniel Dogs with Drug-Resistant Epilepsy
Source: Brain Sci. 2021 Nov 4;11(11):1462. doi: 10.3390/brainsci11111462 (PMC8615928; doi:10.3390/brainsci11111462)
Supplement: Supplementary file 1 [file brainsci-11-01462-s001.zip › brainsci-1446869-supplementary/Suppl File S3_1018.pdf]

brain sciences

Supplementary File S3.

History of the 3 cases before participating in the clinical trial

1. Case 1

A 2 months old, female Cavalier King Charles Spaniel (CKCS) was presented to the Veterinary Medical Teaching Hospital of Nippon Veterinary and Life Science University (VMTH-NVLU) with complaint of recurrent epileptic seizures. The seizures were right facial twitching with salivation and right forelimb clonus, which evolved into generalized tonic-clonic seizures (GTCS). The duration of the seizures was approximately 1 minute. In interictal neurological examinations, blood count, serum chemistry, and urinalysis were unremarkable.

At the age of 3 months, the dog underwent scalp electroencephalogram (EEG), magnetic resonance imaging (MRI), and cerebrospinal fluid (CSF) analysis. Scalp EEG showed bilateral and general synchronized and isolated left frontal interictal epileptiform discharges (EDs). MRI showed slightly small left hippocampus and Chiari-like malformation (CLM; which is a breed specific inherited anomaly in CKCS). CSF was normal. The dog was diagnosed with structural epilepsy of unknown etiology.

At 5 months old, antiseizure medication (ASM) with zonisamide (ZNS) was started due to increasing seizure frequency (> 2 seizures per 3 months). However, the seizures became intractable as the dog matured, and seizure control had not been achieved using multiple ASMs; potassium bromide (KBr) and phenobarbital (PB) had been gradually added. Nevertheless, seizures tended to be severe, and she experienced cluster seizures at 11 months old. At this time, the dog's activity declined and she was mainly asleep during the day.

Follow-up MRI and EEG were performed at 8 months and at 2 years and 2 months of age. MRI showed progressive atrophy of the left hippocampus. EEG showed frequent generalized high-amplitude multiple spikes centered on the frontal and parietal regions.

At the age of 2 years 7 months, the dog developed bilateral facial myoclonic status epilepticus (SE) and was hospitalized at the VMTH-NVLU. Therapeutic coma with continuous intravenous infusion of midazolam, pentobarbital, propofol, and inhalation of isoflurane, intravenous ketamine, and alfaxalone were attempted for more than 24 hours (i.e., super-refractory SE) with EEG monitoring. However, these medications did not terminate SE. Euthanasia was suggested to the owners, but they strongly wished to continue the treatment. After a total of 120 hours, SE was ceased and the dog was safely weaned from therapeutic coma. After discharge from the hospital, the dog was treated with PB (4.7mg/kg; serum concentration, 57 μg/mL; recommended therapeutic range, 10–30 μg/mL), ZNS (7.8mg/kg; serum concentration, 19.6 μg/mL; recommended therapeutic range, 10–40 μg/mL) and KBr (36.5 mg/kg ; serum concentration, 2.4 mg/mL; recommended therapeutic ranges, 0.8–2.0 mg/mL), all at twice a day (BID), and levetiracetam (LEV; 40 mg/kg) thrice a day (TID). However, the frequency of severe bilateral facial myoclonic seizures with occasional GTCS was 10–15 seizures/day, and ataxia and reduced consciousness during interictal period were severe. The owner decided to enter the clinical trial of epilepsy surgery as soon as the recruitment started. Therefore, this dog is the initial case of our clinical trial, and she was 2 years and 9 months old at the time of surgery.

2. Case 2

A 2 years and 3 months old, neutered female CKCS visited a local veterinary clinic due to the onset of epileptic seizure. The dog had GTCS with salivation and urination for approximately 1 min. Physical and neurological examinations, blood count, serum chemistry, and urinalysis were unremarkable. The seizures were recurrent, and the dog underwent EEG, MRI, and CSF analysis at the referral veterinary hospital. The EEG showed interictal EDs, which were sharp waves centered in the mid-occipital region. MRI showed no lesion other than mild CLM. Results of CSF analysis were unremarkable. The dog was diagnosed with idiopathic epilepsy (IE) and started ASM with ZNS (2.8 mg/kg BID). The dog showed GTCS at 1–2 seizures/month, and the dose was increased in stages to 7 mg/kg BID. At 2 years 7 months old, the dog experienced SE and was KBr was added at 20 mg/kg once a day (SID). At 2 years 11 months old, the patient was admitted to the hospital for SE with continuous infusion of midazolam and intravenous LEV. Then, the dose of

ZNS was increased (14 mg/kg BID), and the serum concentration of ZNS was 45.5 μg/mL. The dose of KBr was also increased to 30 mg/kg tBID. Nevertheless, seizure frequency increased to more than 2 seizures/months, and LEV at 20 mg/kg TID was added at 3 years 2 months old. Seizures further increased to 1 seizure/2–3 days and the dose of LEV was increased to 30 mg/kg TID. Around this time, the dog showed wandering behavior as a postictal sign. A second MRI was performed at 3 years 11 months old, but showed no abnormalities. At 4 years old, ASMs were changed to PB (4.7 mg/kg BID) and KBr (30 mg/kg BID) at once, however, PB was discontinued due to severe ataxia and sedation. Then, at age 4 years and 1 month, ASMs were modified with ZNS (6.4 mg/kg BID), LEV (20 mg/kg TID), and gabapentin (GBP; 12 mg/kg BID). After that, the dog's ataxia and sedation improved, but GTCS was observed at 3 seizures/day and drop attacks with head-butting against the cage occurred every 2 hours.

Since the seizures were not improved by ASMs, the dog was referred to the Animal Medical Center of Nihon University. PB (2.2 mg/kg BID) was re-started, but still seizures were not controlled with ZNS (6.9 mg/kg BID; serum concentration, 21.5 μg/mL), PB (2.1 mg/kg BID; serum concentration, 17.2 μg/mL), GBP (13.8 mg/kg BID), and LEV (34 mg/kg TID). In addition to GTCS, bilateral facial myoclonic seizures and circling seizures (running fits) were also observed at approximately 20 seizures/day in total. Most seizures lasted 2- 3 minutes, but at least one seizure per day lasted up to 40 minutes, i.e., the dog had SE every day. During the daytime, the dog was strongly sedated and slept most of the time. Even in the interictal period, the dog could only stand and walk for a short time.

The owners were suggested to enter the clinical trial of epilepsy surgery, and they decided to participate the trial. Then, the dog was re-referred to VMTH-NVLU in order to undergo presurgical evaluations at 4 years 10 months old.

3. Case 3

A 3 years and 4 months old, neutered male CKCS visited a local veterinary clinic with a complaint of the initial seizure. The seizure at the time was GTCS with salivation and urination, and restless and wandering behavior were observed after the seizure. After that, GTCS recurred monthly and the dog was referred to the Japan Animal Referral Medical center of Nagoya at 3 years 10 months old. No abnormalities were found in the interictal neurological examinations and blood tests, but physical examination, thoracic radiograph, and ultrasound of the heart revealed mild mitral regurgitation, which was judged to not require treatment. The EEG showed repetitive interictal EDs, which were spikes and spike-waves from the left fronto-temporal region. MRI was normal except for the presence of CLM. There were no significant abnormalities in CSF. The patient was diagnosed with IE, and ASM was initiated with ZNS (5.6 mg/kg BID; serum concentration, 11.2 μg/mL). Then, GTCS had not been observed for 1 month, but wandering behavior was observed once every 2 days.

At 4 years old, the dog began to exhibit circling 2-3 times a week in addition to sudden wandering. Those behaviors were also suspected as epileptic seizures (i.e., focal behavioral seizures) and the ZNS dose was increased to 11.4 mg/kg BID. However, the seizures were not controlled. Then, PB (3.4mg/kg BID) was started and switched with ZNS. PB monotherapy achieved seizure-free status for 8 months, but the seizures recurred at 4 years 8 months. ASM was modified to PB (7mg/kg BID; serum concentration, 24μg/mL) and KBr (25mg/kg SID). At 5 years 2 months old, the dog presented 2-3 seizures/day and began to show drop attacks (atonic seizures or negative myoclonic seizures) in addition to GTCS. LEV (29.8 mg/kg TID) was added to the PB and KBr.

At 5 years 3 months old, LEV was discontinued due to unchanged seizure frequency, and PB was reduced, but pregabalin (PGB) was added at 2.9 mg/kg BID. After starting PGB, seizures decreased to 1 seizure/day in the morning only. KBr was not changed (25 mg/kg SID), PGB was increased to 2.9mg/kg BID, PB was decreased to 3.7 mg/kg BID, and LEV to 30.5 mg/kg SID (morning only). A 1 month later, LEV was discontinued and PB was reduced (1.9 mg/kg BID) because seizure duration was decreased while seizure frequency was unchanged. At 5 years 1 months old, PB was stopped due to strong somnolence even though seizure frequency was 1 seizure per 3 days; KBr (25 mg/kg SID; serum concentration, 0.4 mg/mL) and PGB (2.9 mg/kg TID) were continued. The seizure frequency declined with KBr (33 mg/kg BID; serum concentration, 1.4 mg/mL) and PGB (2.9 mg/kg TID). However, at 5 years 3 months old, the dog was hospitalized due to CS, and the ASM was again adjusted as KBr (50 mg/kg BID; serum concentration, 1.4 mg/mL), PGB (2.5 mg/kg BID), ZNS (17.4 mg/kg BID; serum concentration, 51.3 μg/mL), and LEV (25 mg/kg TID). After discharge, the seizure frequency was more than 1-2 seizures/day. The dog had ataxia, decreased consciousness, and was not active at all during the daytime. Also, he showed polyphagia and tried to eat the owner's hand offered to him. The dog's status

did not improve even after reducing the dose of ZNS (10 mg/kg BID) and discontinuing PGB, and the seizures became more difficult to control.

Then, an attending veterinarian and the owners applied to enter the trial, the dog was approved as a candidate for epilepsy surgery at 5 years 4 months.
