# Supplementary material for: Corpus Callosotomy in 3 Cavalier King Charles Spaniel Dogs with Drug-Resistant Epilepsy
Source: Brain Sci. 2021 Nov 4;11(11):1462. doi: 10.3390/brainsci11111462 (PMC8615928; doi:10.3390/brainsci11111462)
Supplement: Supplementary file 1 [file brainsci-11-01462-s001.zip › brainsci-1446869-supplementary/Suppl File S4_1103.pdf]

brain sciences

Supplementary File S4.

Methods for each presurgical evaluation

The following is a detailed description of each presurgical evaluation performed at the veterinary medical teaching hospital of Nippon Veterinary and Life Science University. In all cases, scalp electroencephalogram (EEG) under sedation, 3.0 Tesla magnetic resonance imaging (MRI) of the brain, and intraoperative electrocorticogram (ECoG) were performed. In addition, video-intracranial EEG monitoring was performed in Case 1 (before 2nd surgery) and Case 2. In addition, postoperative follow-up scalp EEGs and MRIs were conducted in the same manner as presurgical evaluations described below.

1. Scalp EEG

Scalp EEGs were recorded for approximately 25 minutes under sedation using a digital EEG system (Neurofax EEG-1200; Nihon Kohden, Tokyo, Japan) with synchronous video recording and intravenous administration of dexmedetomidine (10-30 μg/kg). The recording conditions were as follows: sampling frequency, 1,000 Hz; high-cut filter, 60 Hz; time constant, 0.1; and AC filter, on. Exploration subdermal needle electrodes were placed on the frontal (F3, F4), central (parietal) (C3, C4), temporal (T3, T4), and occipital (O1, O2) regions bilaterally (odd = left; even = right) and on 3 midline points (Fz, Cz, Pz, or Oz) [1]. Electrocardiograms were also recorded with the EEG system. Real-time reviewing and recording montages were basically averaged referential and longitudinal bipolar (double banana-like) derivations, but digital re-montages were used in ad lib when the post-recording reviewing.

2. MRI

MRI was performed using a 3.0 Tesla superconducting MRI system (Signa HDxt; GE Healthcare, Tokyo, Japan). General anesthesia was induced by intravenous administration of propofol (7mg/kg) and maintained by inhalation of isoflurane and oxygen. During anesthesia, lactated Ringer's fluid was infused at 5 mL/kg/h. The dogs were positioned in a sternal recumbent position on the table, and the head was placed in a human knee 8-channel array RF coil. The following sequences were acquired: 3D-T2 cube (FSE, TR/TE = 3,200/83 ms, FOV = 15 × 15 cm, slice thickness = 0.6 mm, matrix = 256 × 256, NEX = 1); 3D-T1 SPGR (SPGR, TR/TE/TI = 6.5/3.1/450 ms, FOV = 15 × 15 cm, matrix = 256 × 192, NEX = 1) with and without gadodiamide (0.1 mmol/kg, IV, Omniscan; GE Healthcare); transverse T2-weighted (FSE, TR/TE = 7,000/85 ms, FOV = 15 × 15 cm, slice thickness = 2.0 mm, slice gap = 0.5 mm, matrix = 384 × 288, NEX = 1); T2 fluid-attenuated inversion recovery (FSE, TR/TE/TI = 11,000/140/2,400 ms, FOV = 15 × 15 cm, slice thickness = 2.0 mm, slice gap = 0.5 mm, matrix = 256 × 192, NEX = 2); T2*-weighted (GRE, TR/TE = 740/18 ms, FOV = 15 × 15 cm, slice thickness = 2.0 mm, slice gap = 0.5 mm, matrix = 320 × 192, NEX = 2); diffusion-weighted imaging (DWI; SE propeller, TR/TE = 8,000/94.6 ms, b = 1,000, 3 axes, FOV = 15 × 15 cm, slice thickness = 2.0 mm, slice gap = 0.5 mm, matrix = 128 × 128, NEX = 1); and diffusion tensor imaging (DTI; SE, TR/TE = 8,000/94 ms, b = 1,000, 15 axes, FOV = 15 × 15 cm, slice thickness = 2.4 mm, slice gap = 0.5 mm, matrix = 128 × 128, NEX = 2). Apparent diffusion coefficient (ADC) and fractional anisotropy (FA) maps were created by the internal software of the MRI system. Hippocampal volumetry was performed semiautomatically with the imaging workstation (AZE VirtualPlace Fujin, Canon Medical System, Japan) from 3D-T2 cube data [2].

3. Cerebrospinal fluid (CSF) analysis

Cerebrospinal fluid (CSF) was collected by cisternal puncture from the cerebellomedullary cistern immediately after the preoperative MRI under general anesthesia as described above. CSF (1.5–2.0 ml) was collected from each dog. Collected CSF was examined immediately in house for the following items: color, cell number, cytology, total protein, and some chemical parameters including LDH, AST, CPK, Glu, Ca, micro albumin, Na, K, and Cl.

4. Video-intracranial EEG monitoring

Two dogs (second surgery for Case 1 and Case 2) participated in a long-term (one week) video-intracranial EEG (ViEEG) monitoring to detect laterality and/or localize the seizure-onset zone prior to corpus callosotomy (CC). In Case 1, two custom ECoG electrodes (2.0 cm × 0.5 cm strip-type silicon-sheet with 4 exploration electrodes having 3.0 mm in diameter at 5.0 mm interval; Unique Medical, Tokyo, Japan) were inserted between the longitudinal fissures of the cerebrum, and electrodes were placed in contact with the area around the left cingulate gyrus (**Figure S1A**). In Case 2, a 3 × 3 grid electrode (1.5 cm × 1.5 cm grid-type silicon-sheet with 9 exploration electrodes having 3.0 mm in diameter at 5.0 mm interval; Unique Medical, Tokyo, Japan) were implanted on the dura (epidural space) overlying the parietal lobe in each hemisphere after the craniotomy (**Figure S1B**). The lead from each electrode was fixed on the skull with a titanium plate and screws and on the temporal muscle with sutures, and then passed subcutaneously from the neck skin of the dorsal midline. Bone fragments were re-placed using titanium plates and the head was closed. After the implantation of ECoG electrodes, these dogs were recovered and ViEEG monitoring was performed in a specific monitoring cage for one week. During the monitoring period, disinfection of surgical wounds using gentamicin ointment and administration of antibiotics (cefalexin; 20 mg/kg IV or PO q12h) were conducted. Implanted electrodes were removed at the time of CC.

Continuous ViEEG was monitored within a specific epilepsy monitoring cage for 5-7 days. The inner dimensions of the cage were 70 cm height × 60 cm width × 70 cm depth; five sides, except the front, were made of stainless steel, and the front door was made of acrylic for video monitoring. A 15-channel 360º-rotating slip-ring connector (NSR-15; Neuroscience Inc., Tokyo, Japan) that allowed wired EEG recording in a freely moving animal was attached to the ceiling of the cage. Lead lines from intracranial electrodes were connected to the slip-ring connector via a relay cable long enough for the dogs to move freely in the monitoring cage. The wound through which the electrode wires emerged and the connector between the electrode wires and relay cable were wrapped with gauze and fastened to the neck with an adhesive bandage. The recording conditions were as follows: sampling frequency, 1,000 Hz; high-cut filter, 60 Hz; time constant, 0.1; and AC filter, on. Recorded ViEEG data were stored in the internal hard disk of the EEG system. The example of ictal ViEEG is shown in **Video S1**.

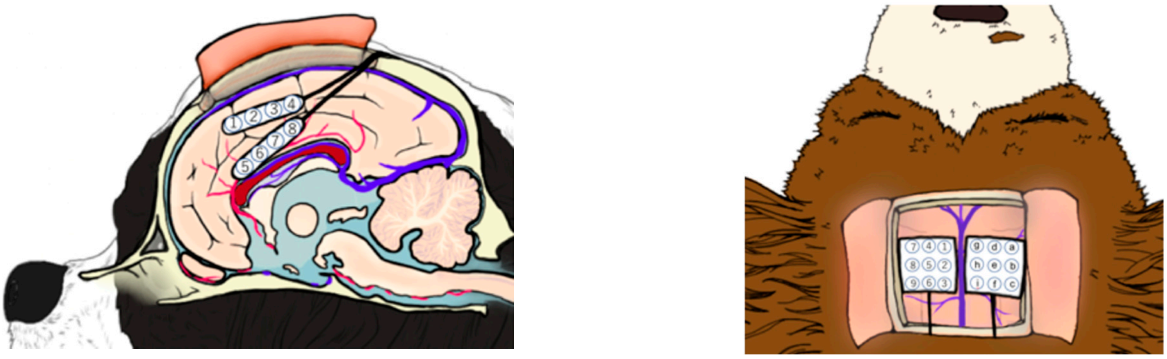

A B

Figure S1. Position of ECoG electrodes for video-intracranial EEG (ViEEG) and intraoperative ECoG. ViEEG monitoring was performed in Case 1 before 2nd surgery (A) and Case 2 (B). Intraoperative ECoG was performed in all cases at pre- and post-corpus callosotomy (A and B).

5. Intraoperative ECoG

During the surgery, intraoperative ECoG was measured in all animals before and after CC. The anesthesia protocol for intraoperative ECoG recording is described in **Supplemental File 5**. First, after the craniotomy, a custom 3 × 3 grid-electrode with one each referral and grand electrode (**Figure S1B**) was placed on the dura matter covering each hemisphere (i.e., 9 electrodes on each hemisphere, total 18 electrodes). Each electrode was covered with moisturized neurosurgical putties during the measurement. After the 5–10 minutes epidural ECoG recording, durotomy and CC were initiated. During dividing hemispheres, one or two 1 × 4 strip-electrodes were used to measure ECoG from the medial

aspect of each hemisphere including the cingulate gyrus and others. Then, after finishing the CC, ECoGs from the medial aspect (as in **Figure S1A**) and surface of each hemisphere (parietal lobe; **Figure S1B**) were measured again to confirm the effect of CC (post-CC intraoperative ECoG). The ECoGs before and after CC are shown for each case in **Figure S2**. The recording conditions intraoperative ECoG were as follows: sampling frequency, 1,000 Hz; high-cut filter, 60 Hz; time constant, 0.1; and AC filter, on.

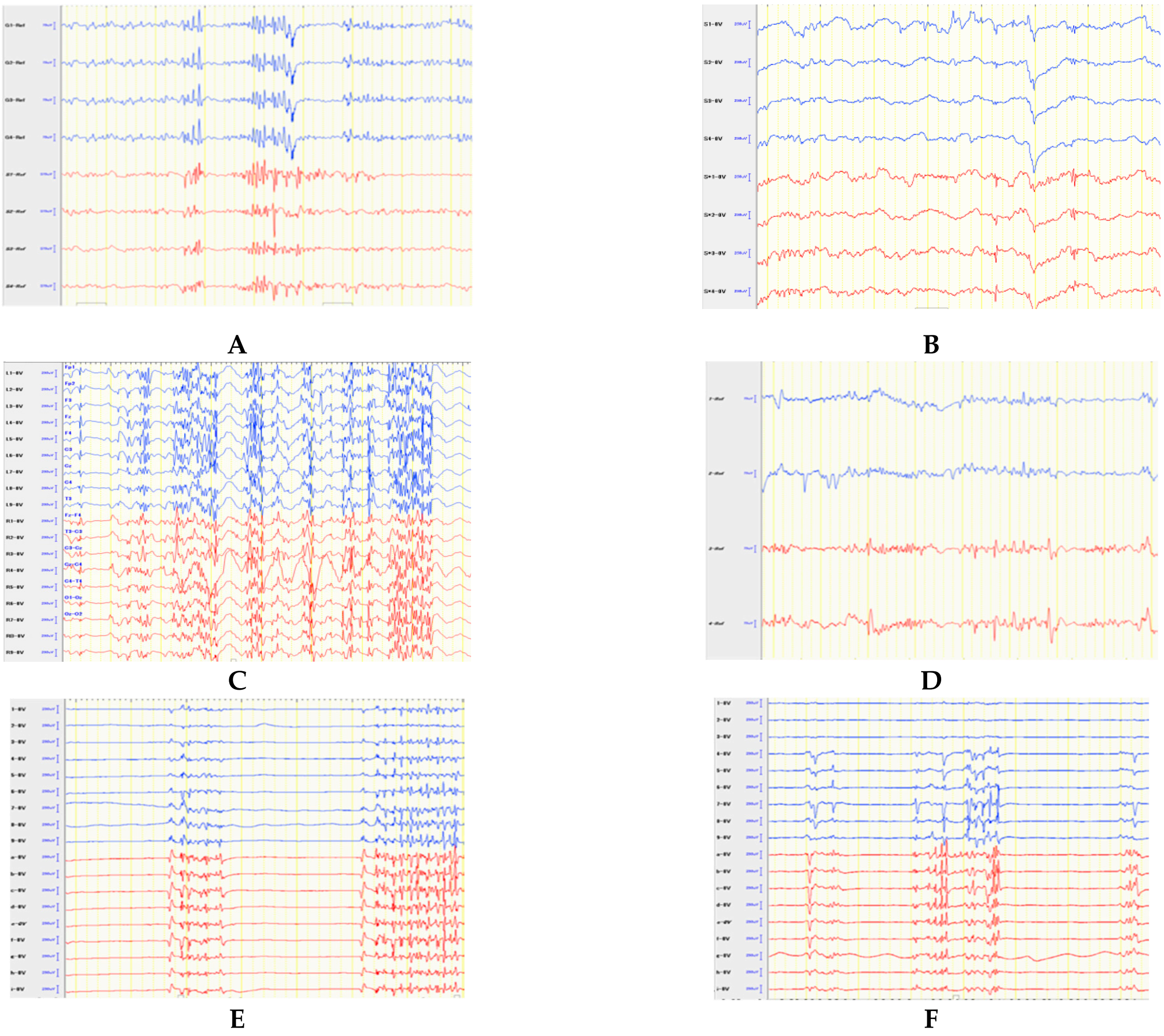

Figure S2. Intraoperative ECoGs before and after corpus callosotomy (CC): Pre (A, C, and E) and post (B, D, and F) CC in Case 1, 2, and 3, respectively. A and B are from a strip electrode placed on the cingulate gyrus of each hemisphere (Case 1). C to F are from a grid electrode placed on each parietal lobe (Case 2 and 3). The montages of ECoG presented correspond to Supplementary Figure 4.1. ECoG was recorded with 2-6 electrodes on the left and right side of brain, depending on the placement of the electrodes on the brain surface.

References

1. Hasegawa, D. Diagnostic techniques to detect the epileptogenic zone: Pathophysiological and presurgical analysis of epilepsy in dogs and cats. *Vet. J.* **2016**, *215*, 64–75, doi:10.1016/j.tvjl.2016.03.005.
2. Mizoguchi, S.; Hasegawa, D.; Kuwabara, T.; Hamamoto, Y.; Ogawa, F.; Fujiwara, A.; Matsuki, N.; Fujita, M. Magnetic resonance volumetry of the hippocampus in familial spontaneous epileptic cats. *Epilepsy Res.* **2014**, *108*, 1940–1944, doi:10.1016/j.eplepsyres.2014.09.009.
