# Supplementary material for: Corpus Callosotomy in 3 Cavalier King Charles Spaniel Dogs with Drug-Resistant Epilepsy
Source: Brain Sci. 2021 Nov 4;11(11):1462. doi: 10.3390/brainsci11111462 (PMC8615928; doi:10.3390/brainsci11111462)
Supplement: Supplementary file 1 [file brainsci-11-01462-s001.zip › brainsci-1446869-supplementary/Suppl File S5_1018.pdf]

brain sciences

MDPI

Supplementary File S5.

Surgical procedures

1. Anesthesia and perioperative medications

All animals were anesthetized with intravenous propofol induction and maintained by continuous rate of infusion (CRI) of propofol (3–6 mg/kg/hr) or inhalation of isoflurane (1.5–2.0%) and oxygen. As an intraoperative analgetic, CRI of fentanyl (2-4 μg/kg/hr) or remifentanil (15-30 μg/kg/hr) was used. At the time of recording intraoperative electrocorticography (ECoG), dose decreasing of propofol-CRI or changing inhalation of isoflurane to sevoflurane was conducted under the fentanyl-CRI. After the operation, CRI of fentanyl was tapered within 12 hours and replaced with a fentanyl patch (25 μg/hr) for 3 days.

During the operation, mannitol (0.5–1.0 g/kg) or concentrated glycerin and fructose (0.5–1.0 g/kg) was infused over 15 minutes to decrease brain swelling as necessary (none to 4 times during the operation). As antibiotics, cefalexin (20 mg/kg, IV) was administered every 2 hours during the operation, and continued with the same dose every 12 hr for 2 weeks after surgery. Antiseizure medications were unchanged from preoperative dose and continued to 12 months (follow-up period) after surgery.

2. Craniotomy

After shaving and sterilization of the head, a midline-centered H-formed scalp incision was made from the level of frontal sinus to 2–3 cm rostral to the external occipital protuberance. Bilateral temporal muscles were reflected from the sagittal crest and detached from the skull. Scalp, subcutaneous tissues, and temporal muscle were retracted bilaterally using Alice forceps and Gelpi retractors. Bilateral (but more widely on the left side) rostro-tentorial craniotomy was performed from the caudal end of the frontal sinus (i.e., not open the frontal sinus) to the rostral end (tip) of the interparietal bone. This longitudinal extend matching to the distance of corpus callosum was decided from preoperative midline sagittal MRI. Two or three burr holes were made on the parietal bone bilaterally, and two (rostral and caudal) transverse-directed slot-like burr holes were carefully made on the midline to visualize underlying dorsal sagittal sinus. Then, each burr hole was connected using a sagittal saw, and the bone fragment was removed. Hemorrhage from the diploic layer of skull was controlled with bone wax, and a pinpoint hemorrhage from a dorsal sagittal sinus (branch to the skull) was stopped with microfibrillar collagen hemostat (Avitene; ZERIA Pharmaceutical, Tokyo, Japan) and/or oxidized cellulose (SURGICEL absorbable hemostat; Johnson & Johnson, Tokyo, Japan). The removed skull fragment was wrapped with gauze soaked with saline and stored until closing the cranium.

3. Intraoperative electrocorticography (ECoG)

During the surgery, intraoperative ECoG was measured in all animals before and after the corpus callosotomy (CC) to confirm the effect of the intervention. Details of ECoG measurement and electrodes are explained in **Supplementary File S4**.

4. Corpus callosotomy (CC)

After the craniotomy and pre-CC ECoG, all intracranial manipulations were performed under the surgical microscope (Leica M525; Leica Microsystems, Tokyo, Japan). Dorsal sagittal sinus-based U-shaped durotomy on the left hemisphere was performed using a no. 11 blade and micro-scissors. Some cortical veins into the dorsal sagittal sinus were cauterized with bipolar forceps. U-shaped dura flap was reversed over to the right hemisphere and covered with neurosurgical putties (SERECEET®; Fuji Systems, Tokyo, Japan) moisturized with saline. The left hemisphere was bluntly

retracted laterally and separated from the falx longitudinally. Small vessels between the cortex and falx were cauterized. Bilateral cingulate gyri were mostly adhesive to each other under the falx, and were separated carefully to visualize the underlying corpus callosum and to recognize the course of artery and vein, i.e., pericallosal artery and vein of the corpus callosum, running along the corpus callosum. Typically, the first incision of the corpus callosum was made at the middle point of the body of the corpus callosum using the bipolar cautery to penetrate to the roof of the third ventricle. Then the bright-whited corpus callosum was longitudinally divided to the genu rostrally and/or to the splenium caudally by repeating ablation with a bipolar cautery and suction with an aspirator. Especially at the tip of the genu and the end of the splenium, it was needed an extremely careful incision to avoid the rostral cerebral artery and the great cerebral vein, respectively. Small bleeding during the CC was controlled by cauterization by bipolar cautery or application of oxidized cellulose. After finishing the CC, post-CC ECoG and MRI were performed to confirm the disconnection of the corpus callosum. Summary of the CC is seen in **Video S2**.

5. Closure

After finishing the procedure of CC and confirming hemostasis in the operating field, dura matter was sutured intermittently with 6-0 absorbent monofilament (PDS Ⅱ, Johnson & Johnson, Tokyo, Japan). If there was a defect not be able to suture, a piece of artifactual dura (dura wave, GUNZE, Osaka, Japan) was applied and sealed with fibrin glue (Beriplast P Combi-Set Tissue Adhesion; CSL Behring, Tokyo, Japan). Small burr holes (2–3 holes each edge) were made on both bone fragment and cranium and those were sutured with 1-0 or 2-0 monofilament polypropylene (Surgipro, Covidien, Tokyo, Japan). In the 2nd surgery of Case 1, cranioplasty was performed with titanium mesh plate and screws. Temporal muscles, subcutaneous tissues, and scalp were closed as usual.

6. Postoperative care

All patients were cared and observed with 24 hours continuous direct or video monitoring in the intensive care unit for 10–14 days after surgery. Postoperative medications were described in the section 1 (Anesthesia and perioperative medications). If the patients showed clinical signs of intracranial hypertension such as decreased consciousness, visual deficit, circling or aimless pacing, concentrated glycerin and fructose (0.5–1.0 g/kg) were infused over 15 minutes adequately. Feed and water are usually restarted 24 hours after surgery. After removing the stitches of the head, the patients were discharged from the hospital.
